# Molecular and biological functions of gingerol as a natural effective therapeutic drug for cervical cancer

**DOI:** 10.1186/s13048-021-00789-x

**Published:** 2021-03-11

**Authors:** Parinaz Zivarpour, Elhameh Nikkhah, Parisa Maleki Dana, Zatollah Asemi, Jamal Hallajzadeh

**Affiliations:** 1 Department of Biological sciences, Faculty of Basic Sciences, Higher Education Institute of Rab-Rashid, Tabriz, Iran; 2 Medicinal Plants Research Cent Maragheh University of Medical Sciences, Maragheh, Iran; 3 Research Center for Biochemistry and Nutrition in Metabolic Diseases, Institute for Basic Sciences, Kashan University of Medical Sciences, Kashan, Iran; 4 Department of Biochemistry and Nutrition, Research Center for Evidence-Based Health Management, Maragheh University of Medical Sciences, Maragheh, Iran

**Keywords:** Cervical cancer, Gynecological cancers, Human papillomavirus, Ginger

## Abstract

Cervical cancer is one of the most common and important gynecological cancers, which has a global concern with an increasing number of patients and mortality rates. Today, most women in the world who suffer from cervical cancer are developing advanced stages of the disease. Smoking and even exposure to secondhand smoke, infections caused by the human papillomavirus, immune system dysfunction and high-risk individual-social behaviors are among the most important predisposing factors for this type of cancer. In addition, papilloma virus infection plays a more prominent role in cervical cancer. Surgery, chemotherapy or radical hysterectomy, and radiotherapy are effective treatments for this condition, the side effects of these methods endanger a person’s quality of life and cause other problems in other parts of the body. Studies show that herbal medicines, including taxol, camptothecin and combretastatins, have been shown to be effective in treating cervical cancer. Ginger (Zingiber officinale, Zingiberaceae) is one of the plants with valuable compounds such as gingerols, paradols and shogoals, which is a rich source of antioxidants, anti-cancer and anti-inflammatory agents. Numerous studies have reported the therapeutic effects of this plant through various pathways in cervical cancer. In this article, we look at the signaling mechanisms and pathways in which ginger is used to treat cervical cancer.

## Introduction

Cervical cancer is one of the most important cancers in women, causing death. Worldwide, this type of cancer ranks fourth in terms of cancer in women. Nearly 85% of cervical cancer deaths are reported in underdeveloped or developing countries. Reports also show that in economically weak or middle-income countries, cervical cancer mortality is about 18% higher than in rich countries. Central and South America, as well as South Asia, are geographically more prone to cervical cancer. In 2016, about 12,990 people were diagnosed with cervical cancer in the United States, of which 4120 died [1]. The average age of these people at the time of diagnosis was reported to be 47 years and half of the diagnoses occur in women under the age of 35 [1, 2]. Smoking, human papillomavirus infection, and immune system dysfunction are risk factors for cervical cancer [3–9]. The results of a cohort study of more than 300,000 women in Europe show that smoking is a major risk factor for cervical cancer [2].

The disease is treatable in its early stages, if diagnosed. Of course, in the long run, the effects of treatment on these people cause other health problems for the person [6]. The choice of cervical cancer treatment depends on the stage of the disease and the location of the tumor. Therapies for this disease include chemotherapy or radical hysterectomy, which may be used in combination. In the early stages of diagnosis and low-risk disease, the person is usually operated on to maintain fertility [2]. In general, chemotherapy is one of the major treatments for cervical cancer today, and vaccination against human papillomavirus (HPV) is one of the preventive measures against this disease [10, 11]. The International Federation of Obstetricians and Gynecologists divides cervical cancer into five stages (I, II, III, IVA and IVB) based on physical examinations and biopsies. In the stage I, although the cancer penetrates from the cervix to the inner layers, it is limited to the uterine tissue itself. The stage II, which is limited to the pelvis, cancer cells are also found in nearby tissues such as the vagina. In the stage III, tumor cells are also found in the lower parts of the vagina and the cancer spreads to the pelvic wall. At this stage, the tumor causes hydronephrosis by affecting the kidney. In the stage IVA, the bladder and rectum are also involved, but the cancer does not affect other parts of the body. In the stage IVB, the cancer spreads to other tissues in the body [12]. Some studies have suggested a combination of chemotherapy, surgery, and radiotherapy to treat cervical cancer. However, treating patients with this method, especially in the advanced stages of the disease, faces some obstacles, including the toxicity of chemotherapeutic drugs, weakness and therapeutic failure [13–16]. Moreover, patients may develop drug resistance which is mostly related to cancer stem-like features, such as diverse receptors and transmembrane proteins (e.g. c-Kit, located on these cells [17]. Therefore, it is very important to adopt alternative therapies with the least amount of side effects for the treatment of cervical cancer and to improve the quality of life of these patients, which requires various studies.

Today, the use of natural ingredients such as herbs and spices are increasing due to their beneficial properties for maintaining human health and their anti-cancer effects. Among the factors that cause various pathological disorders such as neurological, heart and cancer diseases in the body, we can mention the production of oxygen free radicals such as superoxide radical (O2 -), hydrogen peroxide (H2O2). These active oxygen species can also have destructive effects on cells and tissues in the body’s natural metabolism [18, 19]. Medicinal plants and spices are valuable sources of antioxidant compounds due to their phenolic, carotenoid and ascorbic acid factors [18, 20]. Anacardium occidentale in hepatoma, Asparagus racemosus in human epidermoid carcinoma, Boswellia serrata in human epidermal carcinoma of the nasopharynx, Erythrina suberosa in sarcoma, Euphorbia hirta in Freund virus leukemia, Gynandropsis pentaphylla in hepatoma, Nigella sativa in Lewis lung carcinoma, Paederia foetida in human epidermoid carcinoma of the nasopharynx, picrorhiza kurroa in hepatic cancers, and Withania somnifera in various tumors are herbal examples which have anticancer activity [21, 22]. Reports indicate that the use of these plants in the daily diet in South East Asian countries reduces the incidence of breast, prostate, intestinal and other cancers [18, 23]. Studies show that herbal medicines, including taxol, camptothecin and combretastatins, have been shown to be effective in treating cervical cancer [24–26].

Ginger (Zingiber officinale, Zingiberaceae) is one of the oldest herbs used to treat diseases such as colds, coughs, arthritis, digestive disorders, dyspepsia, vomiting, diarrhoea, gastritis, and nausea during pregnancy asthma, inflammation, nervous disease, hepatotoxicity, migraine, diabetes, hypercholesterolaemia, helminthiasis and schistosomiasis. This plant has antibacterial and antifungal properties and is effective in preventing or treating diseases such as cancer due to its antioxidant properties [18, 27–34]. So far, according to the ancient history of the use of ginger in diet, various studies have been conducted on the healing properties of this medicinal plant in a variety of cancers, including cervical, colorectal, pancreatic and breast cancer. In addition to anti-tumor properties, the anti-inflammatory and antioxidant potential of ginger has also been reported in these studies [35–43]. Furthermore, different supplements and gingerol are reported to affect the metabolic profiles [44–48]. In the study of Ansari et al. [18] the successful effects of antioxidant and anti-cancer properties of ginger in controlling cervical cancer and breast cancer has been confirmed [18]. Ginger has active phenolic compounds such as gingerol, paradol and shogoal that show anti-cancer, antioxidant, anti-angiogenic, anti-atherosclerotic and anti-inflammatory potentials [40, 49–53]. Therefore, the prevalence and mortality rate of this cancer is representative of its importance and the need for new diagnostic and therapeutic methods. This article is an attempt to show the therapeutic potential of ginger extract as a natural and herbal compound in the treatment of cervical cancer. This paper also describes the mechanisms of action of ginger compounds in the prevention and treatment of this type of cancer.

### Cervical cancer: prevalence, pathogenesis, and diagnosis

In 2018, with the death of 311,365 women due to cervical cancer, the disease became a global concern [54]. Cervical cancer usually occurs in women with human papillomavirus infection. Studies show that types 16 and 18 of the virus are among the most important risk factors for cervical cancer due to their carcinogenic properties, such as cell gene degradation, increased uncontrollable cell division, and disruption of cell cycle regulation [55]. One of the cases of intrauterine changes is cervical neoplasia, which usually takes several years after the onset to become invasive. Therefore, cervical exfoliation cytology is used to identify intrauterine cervical neoplasia. This procedure can also be used to prevent the development of cervical cancer. As HPV infection became one of the risk factors for developing cervical cancer, preventive vaccination began in the 1990s [1]. So today, vaccination against (HPV) is one of the preventive methods of cervical cancer [10, 11]. Cervical cancer is usually divided into two types of squamous cell carcinoma and adenocarcinoma based on the origin of malignancy [56]. Common screening tests to detect human papillomavirus infection include examination, colposcopy, biopsy, and Papanikolaou smear, which are also used to diagnose cervical cancer [54, 57, 58]. Screening tests such as cytology or Pap smear can be used to identify primary lesions of cervical cancer and to diagnose it early [56, 59]. Due to the wide prevalence of this disease and the complications of the usual treatment methods of this disease that affect patients’ quality of life in the long run, in addition to finding effective biological markers for early diagnosis, finding new and safe treatment methods with the least number of complications. It is very important to improve the effectiveness of treatment and the outcome of the disease.

### Ginger: components

Escape oils (1 to 3%) and oleoresin non-volatile fasteners are ginger compounds. Oleoresin contains a variety of active ingredients, including gingerols and shogaols. Gingerols are homologous compounds that have different non-branched alkyl chains. Shogaols are also homologues that are derived by gingerols dehydration at C4 and C5. These compounds are produced when long-term ginger is stored or heated [35, 36, 60–62]. Therefore, most of the fresh ginger compounds are Gingerols compounds, especially 6-gingerol. Shogaols are found largely in dried ginger [35, 60, 62, 63]. 6-gingerol, 8-gingerol, 10-gingerol and 6-shogaol are the most important and major compounds in Oleoresin [39, 64]. In general, gingerols are the most common compounds found in ginger, and reports indicate that these compounds have a variety of therapeutic potentials, including tumor prevention [39, 65–69], antipyretic [39], pain-reducing [70], and cardiotonic [71] effects. These compounds also have anti-inflammatory, anti-angiogenic, antibacterial and antifungal properties and reduce high blood cholesterol levels [35, 38, 72, 73]. Other compounds found in ginger include waxes, fats, vitamins, minerals and carbohydrates. Its rhizomes also produce a proteolytic enzyme called zingibain [40].

### Ginger: pharmacokinetics

Numerous clinical studies to evaluate the pharmacological and therapeutic effects of ginger have not measured the amount of active ingredients in ginger extract. Therefore, different results from clinical trials can be generalized to the lack of use of the standard dose of ginger extract [35, 74].

Although extensive clinical trials have been conducted on the pharmacological effects of ginger, sufficient studies on the pharmacokinetics of ginger active compounds in human biological systems are not available. Therefore, concentrations of 6-, 8- and 10- gingerol and 6- shogaol for their effectiveness in the body have not yet been fully obtained.

### Effects of ginger in cervical cancer (clinical studies)

Various studies have been conducted on the effects of ginger on a variety of cancers, including cervical cancer. The results of all research agree on the anti-cancer properties of this plant. Reports attribute the anti-cancer potential of ginger to its inhibitory effects on cell proliferation and induction of apoptosis [13, 18, 75, 76]. 6-gingerol as the most important functional component in ginger has a strong anti-cancer and anti-inflammatory potential [77]. 6-gingerol through inhibition of IkBα, nuclear translocation of NF-kB, suppression of Inducible nitric oxide synthase (iNOS), release of cytochrome c, increased expression of Apoptotic protease activating factor 1(Apaf-1), activation of caspase, stimulation of oxidative stress, induction of DNA damage, autophagy and increased protein activity of p53 and p21, causes apoptosis and thus prevents cancer progression and tumor growth [10, 78–84].

Another species of ginger that has been known for its thin white skin, fragrant juice, and crispness for more than a thousand years is known as Tongling White Ginger. This plant is welcomed as one of the best ginger species in China [85, 86]. The reports confirmed the valuable properties of another important ingredient in ginger extract called 10-gingerol, including anti-cancer, anti-inflammatory and antioxidant [85, 87, 88]. 6-Shogaol is another important biologically active ingredient in ginger that has anti-cancer properties. Various pathways including p38 mitogen-activated protein kinase, extracellular signal-regulate kinase 1/2 and c-Jun N-terminal Kinase 1/2, phosphatidylinositol 3-kinase/Akt and cell cycle checkpoint proteins cdk1 and cyclin B and cdc25C are involved in cell death induced by 6-Shogaol in cancer cells [16, 89, 90]. However, the pathways of apoptosis induction and cell signaling cascades that 6-Shogaol has in the removal of cervical cancer cells have not been fully investigated [16, 91]. Naturalness, antioxidant potential, easy access to the environment, easy metabolism and low cost of ginger have made this plant a natural remedy for effective chemotherapy in various cancers. However, there are not enough studies on its exact anti-cancer effects and its functional mechanism in improving cervical cancer. Therefore, in this part of the article, we summarize the mechanisms and signaling pathways of this highly effective medicinal plant in cervical malignancy. These signaling pathways are summarized in (Table 1).

**Table 1:** Experimental studies of gingrol in cervical cancer

Form of resveratrol	Doses	Problem	Model	Findings	Ref
(Ginger) 6-gingerol	50 μM for 24 h	Cervical cancer caused by the human papillomavirus	*In vitro* [78]	Stopping the progression of cervical cancer by stimulating cell proliferation inhibition and induction of apoptosis	[10]
(Ginger) 6-gingerol	50 μM for 24 h	Cervical cancer caused by the human papillomavirus	*In vitro* [78]	Stopping the progression of cervical cancer by inducing p53-dependent apoptosis independent of HPV oncoproteins (E6 and E7)	[10]
(Ginger) 6-gingerol	50 μM for 24 h	Cervical cancer caused by the human papillomavirus	*In vitro* [78]	Stopping the progression of cervical cancer by stimulating reactivation of p53 through proteasome inhibition	[10]
(Ginger) 6-gingerol	50 μM for 24 h	Cervical cancer caused by the human papillomavirus	*In vitro* [78]	Stopping the progression of cervical cancer by stimulating the production of ROS, DNA damage and reactivating p53	[10]
(Ginger) 6-gingerol	50 μM for 24 h	Cervical cancer caused by the human papillomavirus	*In vitro* [78]	Stopping the progression of cervical cancer by enhancing the anti-proliferative properties of cisplatin	[10]
(Ginger) 6-gingerol	2 and 5 mg/kg bodyweight	Cervical cancer caused by the human papillomavirus	*In vivo (mice)*	Stopping the progression of cervical cancer by stimulating cell proliferation inhibition and induction of apoptosis	[10]
(Ginger) 6-shogaol	15 μM for 24 h	Cervical cancer	*In vitro* [78]	Stopping the progression of cervical cancer by standing the cell cycle at G2 / M stage through mitochondrial pathways and endoplasmic reticulum stress	[16]
(Ginger) 1′S-1′-acetoxychavicol acetate or ACA)	20 μM for Ca Ski and 30 μM for SiHa for 12 h plasmid transfection or 48 h miRNA transfection	Cervical cancer	*In vitro (Ca Ski and SiHa)*	Stopping the progression of cervical cancer by stimulating apoptosis by inhibiting miR-629 expression and increasing RSU-1 expression conditions in cardiac fibroblasts	[13]
Zingiber cassumunar Roxb	56.12 + 0.21 μg/ml cytotoxicity, 7.45 + 0.01 μg/ml PGE2 inhibitor	Women’s Health Remedy (cevical cancer)	*In vitro* [78]	Stopping the progression of cervical cancer by Cytotoxicity function and suppressing cell proliferation by inhibiting the production of prostaglandins	[92]
Zingiber officinale Roscoe	42.07 + 2.01 μg/ml cytotoxicity, 4.78 + 1.60 μg/ml PGE2 inhibitor	Women’s Health Remedy (cevical cancer)	*In vitro* [78]	Stopping the progression of cervical cancer by Cytotoxicity function and suppressing cell proliferation by inhibiting the production of prostaglandins	[92]
Zingiber zerumbet (Linn) Smith	4.42 + 0.20 μg/ml cytotoxicity, 11.34 + 0.28 μg/ml PGE2 inhibitor	Women’s Health Remedy (cevical cancer)	*In vitro* [78]	Stopping the progression of cervical cancer by Cytotoxicity function and suppressing cell proliferation by inhibiting the production of prostaglandins	[92]
Alpinia pahangensis	1, 10, 50, 100 μg/ml for 72 h	Cervical cancer	*In vitro (Ca Ski)*	Stopping the progression of cervical cancer with antioxidant and cytotoxic properties	[76]
Zingiber officinale	12.5, 25, 50 and 100 μg/mL for 24 h	Cervical and breast cancers	*In vitro* [78]	Stopping the progression of cervical cancer with antioxidant and cytotoxic properties	[18]
Tongling White Ginger (10-gingerol)	30 μM for 60 h	Cervical cancer	*In vitro* [78]	Stopping the progression of cervical cancer by inducing apoptosis through altering cell morphology	[85]
Tongling White Ginger (10-gingerol)	30 μM for 60 h	Cervical cancer	*In vitro* [78]	Stopping the progression of cervical cancer by stanging the cell cycle in stage G0 / G1	[85]
Tongling White Ginger (10-gingerol)	30 μM for 60 h	Cervical cancer	*In vitro* [78]	Stopping the progression of cervical cancer by stimulating apoptosis	[85]
Tongling White Ginger (10-gingerol)	30 μM for 60 h	Cervical cancer	*In vitro* [78]	Stopping the progression of cervical cancer by inhibiting cell proliferation by suppressing the PI3K / Akt pathway	[85]

### 6-gingerol improves cervical cancer caused by human papillomavirus by suppressing cell proliferation and stimulating cell apoptosis

The results of a study conducted by Rastogi et al. [10] in both *in vivo* on mice and *in vitro* on Hela cells show that the administration of 50 μM 6-gingerol, significantly inhibited the growth and proliferation of cervical cancer cells. Cellular and tissue studies show that ginger increases the percentage of apoptotic cells in both the initial stage of apoptosis and its final stage [10]. Therefore, ginger (6-gingerol) inhibits cell proliferation by inducing apoptosis cell death and can help control the progression of cancer.

### 6-gingerol improves cervical cancer by reactivating p53 without inhibiting HPV oncoprotein

Disabling the transcription of HPV oncoproteins (E6 and E7) inhibits the proliferation of cervical cancer cells. Many natural drug compounds demonstrate their anti-cancer potential by inhibiting these E6 and E7 proteins [10, 93, 94]. 6-gingerol stimulates both p53-dependent apoptosis and p53-independent apoptosis in cancer cells. P21 is the functional mediator of p53-dependent apoptosis pathways [10, 83]. Evaluations by Rastogi et al. [10] show that after administration of 6-gingerol, p21 mRNA expression levels in Hela cells increase significantly. These findings suggest that in these cells, the 6-gingerol-induced apoptosis is of the p53-dependent apoptosis type [10]. Therefore, it can be said that 6-gingerol reactivates p53 and increases the level of P21 with the onset of p53 activity. This process occurs under the influence of ginger without the need for suppression of E6 transcription in cervical cancer cells [10].

### 6-gingerol inhibits the progression of cervical cancer by suppressing proteasome and reactivating p53

In cervical cancer cells, p53 endogenous levels are low in the presence of human papillomavirus infection because in these cells the E6 and E6-AP proteins cause the rapid destruction of p53 by proteasomals [95]. In these cells, for the reactivation of p53, transcription and translation of the E6 protein must be inhibited or proteasome activity stopped using inhibitors. In this way, p53 levels and activity are indirectly restored [96–98]. In cervical cancer cells, 6-gingerol has not been reported to affect E6 and E7 mRNA levels, but it does increase p53 and p21 expression. Under these conditions, the level of apoptosis in cancer cells increases and their proliferation stops [10].

Studies show that in most human malignancies, including cervical cancer, the p53 tumor suppressor is inactivated. In cervical cancer, oncoprotein E6 inactivates p53. Oncoprotein E6 binds to E3 ubiquitin ligase E6-AP, thus inducing proteosomal degradation of P53 [10, 95]. Therefore, reactivation of p53 is one of the effective therapeutic goals in improving cervical cancer. Inhibition of proteosomal degradation of p53, suppression of viral protein expression or their inactivation are among the methods of reactivation of this tumor suppressor protein [10, 99, 100]. Proteasomes are involved in the non-lysosomal degradation of intracellular proteins [10, 101, 102]. Various cancers, including cervical cancer, can occur as a result of adverse activation of proteosomes [103, 104]. Some studies show that increased proteasome activity stimulates cancer progression by stimulating survival pathways and drug resistance in tumor cells [10, 102]. Therefore, the main strong anti-cell proliferation function of ginger *in vivo* is due to proteoosmal inhibition and reactivation of p53, which inhibits the growth and development of cervical cancer by inhibiting cell proliferation and stimulating apoptotic cell death.

### 6-gingerol in cancer cells, including cervical cancer, can cause DNA damage by producing ROS, which slows down the progression of cervical cancer by activating p53 in response to DNA damage

Reactive oxygen species (ROS) in cancer cells are caused by functional changes in cytochrome p450, iNOS, Nox NADPH oxidases, decreased antioxidant protein activity, or inhibition of mitochondrial respiratory complex I (MRC I) [10, 84]. Studies show that in cancer cells, including cervical cancer cells, 6-gingerol inhibits MRC I activity, thereby producing ROS. Accumulation of ROS in the cell activates multiple signaling pathways [10, 77, 84]. Thus, by suppressing MRC-I activity in cervical cancer cells, 6-gingerol increases the production of active oxygen species, which in turn causes oxidative stress and DNA damage. Therapies that produce reactive oxygen species are important factors in inducing apoptosis in cancer cells [84]. Studies show that proteasome inhibitors increase the level of ROS accumulation in cancer cells [105].

In Rastogi’s study, it was shown that in cervical cancer cells, 6-gingerol inhibits the activity of proteosomes by increasing the accumulation of reactive oxygen species and causes oxidative stress [10]. The occurrence of such processes causes further activity of p53 and p21, which responds to DNA damage [10, 106] and, by stopping the cell cycle, causes apoptosis of the cancer cell [10]. Harmful and irreversible DNA damage interrupts cell proliferation and causes the cell cycle to stop at S or G2 / M stages [10, 107]. Therefore, 6-gingerol stimulates the production of ROS, causing oxidative DNA damage in cancer cells in the cervix, which then stops the cell cycle in the G2 / M stage. In cancer cells, p21 is considered to be one of the important functional mediators of apoptosis related to p53 [108]. The results of this study also show that reactivation of p53 and apoptosis through proteasomal inhibition is one of the leading roles of 6-gingerol in cervical cancer cells caused by HPV, and stimulation of ROS production is a support mechanism in this cells [10]. In many studies, the process of producing reactive oxygen species has been confirmed as a natural mechanism supporting cancer cell death [10, 109, 110].

### 6-gingerol supports the inhibitory effects of cisplatin on cell proliferation

One of the most effective and practical chemotherapy drugs in the treatment of various cancers, including cervical cancer, is cisplatin. The effects of this drug are shown by prescribing high doses, which causes severe and undesirable side effects in the patient [10, 111, 112]. Numerous studies on HeLa cells have shown that combination therapy with natural agents and cisplatin can reduce the dose of this drug with the same effects as before [10, 113]. In Rastogi’s study, after using a combination of 6-gingerol and cisplatin on cervical cancer cells, the level of production of reactive oxygen species was examined [10]. The results show that such combination therapy further increases the levels of ROS production in these cells. Under these conditions, the levels of oxidative stress applied to the cancer cells also increases. On the other hand, an assessment of H2AX (H2A histone family member X) phosphorylation levels shows that DNA damage is further enhanced by this combination therapy, and the cell cycle in most cells stops at the G2 / M stage [10]. The study also found that *in vivo* the Ki67 cell proliferation marker in cervical cancer cells was significantly reduced by the combined treatment of 6-gingerol and cisplatin compared with their single use. These cells are also more prone to apoptotic TUNEL formation [10]. Therefore, it can be found that combining ginger with cisplatin by increasing oxidative stress, DNA damage, and stimulating cell death in cervical cancer cells supports the anti-cell proliferation effect of cisplatin.

### 6-shogaol inhibits the progression of cervical cancer by stimulating apoptosis and stopping the cell cycle at G2/M stage through mitochondrial pathways and endoplasmic reticulum stress

6-shogaol stimulates apoptosis cell death through mitochondrial pathways and endoplasmic reticulum (ER) stress and stops the cell cycle in G2 / M stage. In this condition, the potential of mitochondrial membrane of cervical cancer cells is disturbed and progression of cervical cancer is prevented [16]. Liu et al. [16] to investigate the effect of 6-shogaol,ginger composition, on cell death in human cervical cancer cells in culture medium, mitochondrial apoptosis-related proteins levels such as pro caspase-3, poly ADP ribose polymerase (PARP), and apoptosis regulator Bax, as well as endoplasmic reticulum stress-related proteins levels., including protein kinase RNA-like endoplasmic reticulum kinase (PERK), CHOP, ARF5, and HSP60, were evaluated [16]. The results show that 6-shogaol reduces the expression of PERK and ARF5 proteins but increases the expression of HSP60. 6-Shogaol does not alter the expression of CHOP. Therefore, in cervical cancer cells, ER stress due to administration ginger plays an important role in the apoptosis of these cells [16].

The role of Bcl-2 family members is crucial in regulating the process of apoptosis [16, 114]. Bax protein is a member of this family [114], and studies show that the expression of this protein increases under the influence of 6-shogaol [16]. Caspase proteins play an important role in initiating the process of apoptosis. Caspase-3 is the most important executive protein of apoptosis, which is activated by caspase-9, the upper caspase inhibitor, during the internal path of apoptosis. Reportedly, 6-shogaol increases the activity of caspase-3. The PARP enzyme, which is a DNA repair enzyme, is the downstream target of caspase-3 and 7. The gap that PARP creates depends on the type of apoptotic responses [16, 115]. Studies show that 6-shogaol causes a breakdown in the structure of the PARP enzyme and inactivates it [16]. Thus, in cervical cancer cells 6-shogaol stops the cell cycle in the G2 / M stage and significantly inhibits the growth of these malignant cells by pushing the cells toward cell death and apoptosis. It also activates various signaling cascades in the apoptosis pathway by activating Bax, caspase-3 proteins and destroying the structure of PARP enzyme, and ultimately destroys the potential of mitochondrial membrane.

### 1′S-1′-acetoxychavicol acetate (ACA) in ginger stimulates apoptosis in cervical cancer cells by inhibiting miR-629 expression and increasing RSU-1 expression

1′S-1′-acetoxychavicol acetate or ACA is another compound found in wild ginger Alpinia conchigera. Studies show that this compound is effective in eliminating cancer cells such as cervical cancer [13, 116, 117]. MicroRNAs are small non-coding molecules that are responsible for regulating genes after transcription [118]. Studies show that in many cancers, the activity of these molecules is disrupted, affecting the regulation of cellular mechanisms such as apoptosis, cell proliferation, metastasis, and sensitivity to chemical reagents [13, 119–123]. MiR-629 is a member of miRNAs that is expressed in a variety of cancers, including breast, cervical, lung, bladder, kidney, and uterine cancers [124]. Studies show that lung and gastric cancer can be detected by assessing the increased levels of this molecule in the bloodstream [13, 125, 126].

Clinical reports indicate that in cervical cancer cells, the level of expression of miR-629 decreases with the presence of ACA. By inhibiting this molecule, cell proliferation is suppressed and the cells are directed to apoptosis [13, 127]. In cervical cancer cells infected with the human papillomavirus, the level of miR-629 expression is very high. The reason for the high level of this molecule is attributed to the expression of E6 / E7 in these cells [13, 128–130]. The miR-629 can be directly connected to the Ras suppressor-1 (RSU-1) and adjust the negative level of its expression so that increasing the miR-629 reduces the level of the RSU-1 and vice versa [13]. The findings are confirmed in a study by Phuah et al. [13] on cervical cancer cells. In their study, they found that MiR-629 expression was inhibited by ACA (a compound in ginger) prescription. As a result, the level of RSU-1, which is involved in inhibiting cell proliferation and stimulating apoptosis, increases. Therefore, ginger through the above pathway can inhibit the growth of cervical cancer cells and destroy them. Further expression of RSU-1 enhances the properties of ACA in inhibiting cell growth and stimulating cell death [13].

### Ginger prevents the proliferation of cancer cells by inhibiting the production of prostaglandins and improves cervical cancer

Prostanoids are involved in processes such as the proliferation and differentiation of cancer cells, suppression of apoptosis, and the development of malignancies. Prostaglandin D2 (PGD2), Prostaglandin E2 (PGE2), prostaglandin F2 (PGF2), prostaglandin I2 (PGI2) and thromboxane A2 are the most important types of prostanoids [131, 132]. Prostaglandins are important factors involved in the progression of cancer and malignancy and the process of angiogenesis. Prostaglandin E2 has been shown to play an important role in signaling pathways that stimulate angiogenesis, metastasis, tumor cell growth, and inhibition of apoptosis [133]. Prostaglandins are derived from arachidonic acid by the activity of the enzyme Cyclooxygenase (COX). These molecules are involved in causing cancer, inflammation and other pathophysiological processes. The COX2 enzyme is activated in response to inflammation and cell growth regulation. Therefore, it can be said that the use of natural medicinal plants that can inhibit the production of PGE2 can prevent cancer and inflammation [92]. Jaiaree and colleagues [92] in the study of cervical cancer cells found that herbal compounds, including Zingiber cassumunar Roxb, Zingiber officinale Roscoe and Zingiber zerumbet (Linn) Smith, used in Thai traditional medicine, reduce the level of Prostanoids such as PGE2, thereby showing their anti-cancer and anti-inflammatory effects, and improve cervical cancer [92].

### Ginger with its antioxidant and cytotoxic properties prevents and treats cervical cancer

In the human body, free radicals such as reactive oxygen species (ROS), are produced by aerobic respiration and oxidative compounds, leading to oxidative stress. Reports indicate that these compounds are involved in diseases such as diabetes, cancer, Parkinson’s, Alzheimer’s, aging, and atherosclerosis [18, 76, 134–138]. Studies show that in normal cells, the concentration of active oxygen species causes gene mutations and disrupts cell signaling pathways and the balance of transcription factors. As a result, the cells are severely damaged and lead to cancer [18, 139]. Other external factors, such as lifestyle and diet, increase the production of free radicals in the human body, in which case the body’s natural antioxidant systems will not be able to clear active species. Under these conditions, important cellular molecules, including phospholipids, carbohydrates, DNA and proteins, are severely damaged and destroyed by oxidative stress. By stimulating the activity of the body’s endogenous antioxidant enzymes and using natural antioxidant compounds, it is possible to move towards reducing oxidative stress [18, 76, 140–142]. Today, a number of studies have focused on the valuable antioxidant properties of natural phenolic compounds, which has led researchers to explore and study different types of these plant-derived compounds.

Various studies on Alpinia pahangensis and Zingiber officinale Roscoe from the ginger family have shown that these plants have antioxidant potential in addition to their antibacterial, antifungal and other properties [18, 76]. Alpinia pahangensis is a rare wild species of ginger found in Pahang and Malaysia [76]. In a study of cervical cancer cells, Phang et al. [76] concluded that treating normal healthy cells with Alpinia pahangensis prevents cancer. They found that methanol and ethyl acetate extracts in this plant are natural sources rich in antioxidants. On the other hand, Phang and his [76] studied the effect of Alpinia pahangensis on cervical cancer cells and found that this plant with its cytotoxic and antioxidant properties can cause the death of these cancer cells [76]. In another study by Ansari et al. [18] on the effect of Zingiber officinale on breast cancer and cervical cancer cells, they found that the plant’s methanolic extract, with its anti-cancer and antioxidant potential, prevents cell proliferation and cell colonization. The results of their study show that in addition to directing the cells to apoptosis, the methanolic extract in this plant also changes the nucleus morphology of these cells, which destroys the cells. Therefore, this plant can be used to prevent, manage and treat cervical and breast cancer [18].

### 10-gingerol inhibits cervical cancer by altering cell morphology

Morphological study of cervical cancer cells after treatment with 10- gingerol shows that this combination reduces the number of cells and the cells go out of their normal state and lose their connections with other cells. Also, the apoptotic bodies are observed and in the cytoplasm of these cells, after treatment with 10- gingerol, many vacuoles appear. All these findings show that this combination eliminates cervical cancer cells by making changes in the morphology and shape of these cells [85].

### 10. Gingerol stops the progression of cervical cancer by stopping the cell cycle in stage G0/G1

Reportedly, 10-gingerol suppresses the cell cycle in G0/G1. Examination of the expression of several important genes related to the cell cycle, including CDK-1, CDK-2, CDK- 4, CDK-6, cyclin A, cyclin B1, cyclin D1, cyclin E1, GSK-3β, β-catenin, and p15, p16, p21, p27 shows that the expression of CDK-2, CDK-4, CDK-6, cyclin A, cyclin D1, cyclin E1, p15 and p21 mRNA significantly decreases in the presence of 10-gingerol [85]. A slight decrease in expression of CDK-1, GSK-3β, β-catenin, and p16 and p27 mRNA is also seen. Cyclin A and cyclin D1 are the most important markers of the G0 / G1 stage of the cell cycle [85]. A study by Zhang et al. [85] shows that 10-gingerol significantly reduces the level of expression of these markers. Thus, 10-gingerol controls the progression of cervical cancer by affecting the expression of cell cycle-related marker genes at both transcription levels and after transcription [85].

### 10-gingerol inhibits cervical cancer through apoptosis

Apoptosis is a regulated biological process of cell death that has two main pathways: the outer and inner pathways [85, 143]. At the cell membrane surface, there are cell death receptors 3 and 5 (DR3 and DR5) as receptors for tumor necrosis factor. These receptors mediate the process of apoptosis and differentiation [85, 144]. 10-gingrol significantly increases the expression levels of apoptotic proteins (DR3 and DR5). As the expression of these proteins increases, apoptotic indicators, such as caspases − 3, − 8, and − 9, are activated and trigger the caspases cascading. By launching these cascades, 10-gingrol induces apoptosis in cervical cells, and thus can play an important role in the treatment of cervical cancer [85]. Other studies have confirmed the increase in caspase-3 expression by 10-gingrol in cervical cancer cells [145]. To investigate the effect of ginger on the mitochondrial pathway, Zhang and his colleagues [85] evaluated the expression of, Bad, Bid, Bax, Bcl-2 and cytochrome c. They found that the expression of these proteins, except Bcl-2, increased in the presence of 10-gingrol. With a slight decrease in Bid, the level of Bcl-2 decreased significantly, which eventually led to the activation of Bax. Therefore, 10-gingrol activates apoptosis signaling pathways and causes mitochondrial dysfunction in cervical cancer cells, causing cell death and inhibiting cervical cancer [85].

### 10-gingrol inhibits the proliferation of cervical cancer cells by inhibiting the PI3K / Akt pathway

PI3K/Akt pathway, which is upstream signaling pathway of the mTOR, is one of the most important factors in regulating cancer progression. The proteins in this pathway are activated by phosphorylation at specific sites [85, 146]. Zhang and his colleagues [85] showed in their study that signaling the PI3K/Akt pathway is another case in which 10-gingrol could affect its anti-cancer potential. Suppression of PI3K/Akt signaling can stop the cell cycle and prevent cell proliferation in cervical cancer cells [85]. The results of this study also show that PI3K causes changes in protein kinase Cε (PKCε) expression and decreases nuclear factor-Kappa B (NF-kB) expression [85, 147]. By stimulating AMPK activity, 10-gingrol reduces the level of phosphorylation of the mTOR pathway and inactivates it [85]. Thus, by inhibiting cell proliferation, 10-gingrol induces cell death in cervical cancer cells.

### Signaling pathways that may be involved in ginger effects on cervical cancer

As we mentioned before, ginger plays a variety of anti-tumor roles in different cancers. There are some studies on cancers other than cervical cancer that deal with ginger effects on certain signaling pathways. Although currently there is no study investigating these signaling pathways in cervical cancer, they may be involved in ginger effects on this cancer.

The Nuclear Factor Kappa B (NF-kB) family is consisted of transcription factors involved in inflammation and immune responses [148]. Studies have shown that NF-kB plays different roles in initiation of cancers as well as their progression and drug-resistance. This signaling pathway that is stimulated by HPV infection exerts important roles in cervical cancer. NF-kB induces the transcription of genes involved in proliferation (e.g. c-myc and cyclin D1), VEGF-dependant angiogenesis, metastasis, and telomerase-dependent cell immortality. Furthermore, activation of NF-kB leads to the expression of cytodine deaminase and APOBEC proteins, leading to cervical cancer’s mutagenic properties [148]. [6]-gingerol treatment leads to the downregulation of the extracellular signal-regulated kinase (ERK) pathway in human pancreatic duct cell-derived cancer cell line PANC-1 [149]. Subsequently, NF- κ B/Snail nuclear translocation is suppressed. Also, it is concluded that [6]-gingerol treatment suppresses invasion and metastasis through NF- κ B/Snail inhibition [149]. Ginger extract is reported to significantly inhibit the activation of NF-κB in ovarian cancer cell lines, CaOV3 and SKOV3 [150]. Activator protein 1 (AP-1) is a transcription factor involved in HPV-mediated cervical carcinogenesis and chemo-radio-resistance. Besides, AP-1 activity and expression loss has been associated with a reduction in the viability and proliferation of UV-irradiated non-stem cervical cancer cells [151]. Oral administration of [6]-Shogaol inhibits phosphorylation of IκB, c-jun and c-fos; leading to the suppression of p65, NF-κB, and AP-1. Consequently, suppressing the activation of NF-κB and AP-1 leads to the inhibition of inflammation and cell proliferation in hamster buccal pouch carcinogenesis [152]. Ling et al. [153] has also reported that 6-shogaol reduces the transcriptional activity of NF-κB in MDA-MB-231 breast cancer cells. Furthermore, they indicated that 6-shogaol inhibits the activation of JNK without reducing the transcriptional activity of AP-1 [153]. Signal transducer and activator of transcription 3 (STAT3) is a transcription factor involved in cellular proliferation, survival and differentiation. STAT3 has attracted a lot of attention as a cancer therapeutic target in several cancers, such as ovarian cancer, neck squamous cell carcinoma, and cervical cancer [154]. In hepatocellular carcinoma HepG2 cells, 10-gingerol inhibits the activation of Src and STAT3 and suppress proliferation [155]. A study on colorectal cancer has also demonstrated that 8-gingerol inhibits epidermal growth factor receptor (EGFR) signaling. Moreover, it suppresses proliferation and migration by EGFR/STAT/ERK axis [156].

## Conclusions

**Fig. 1:**
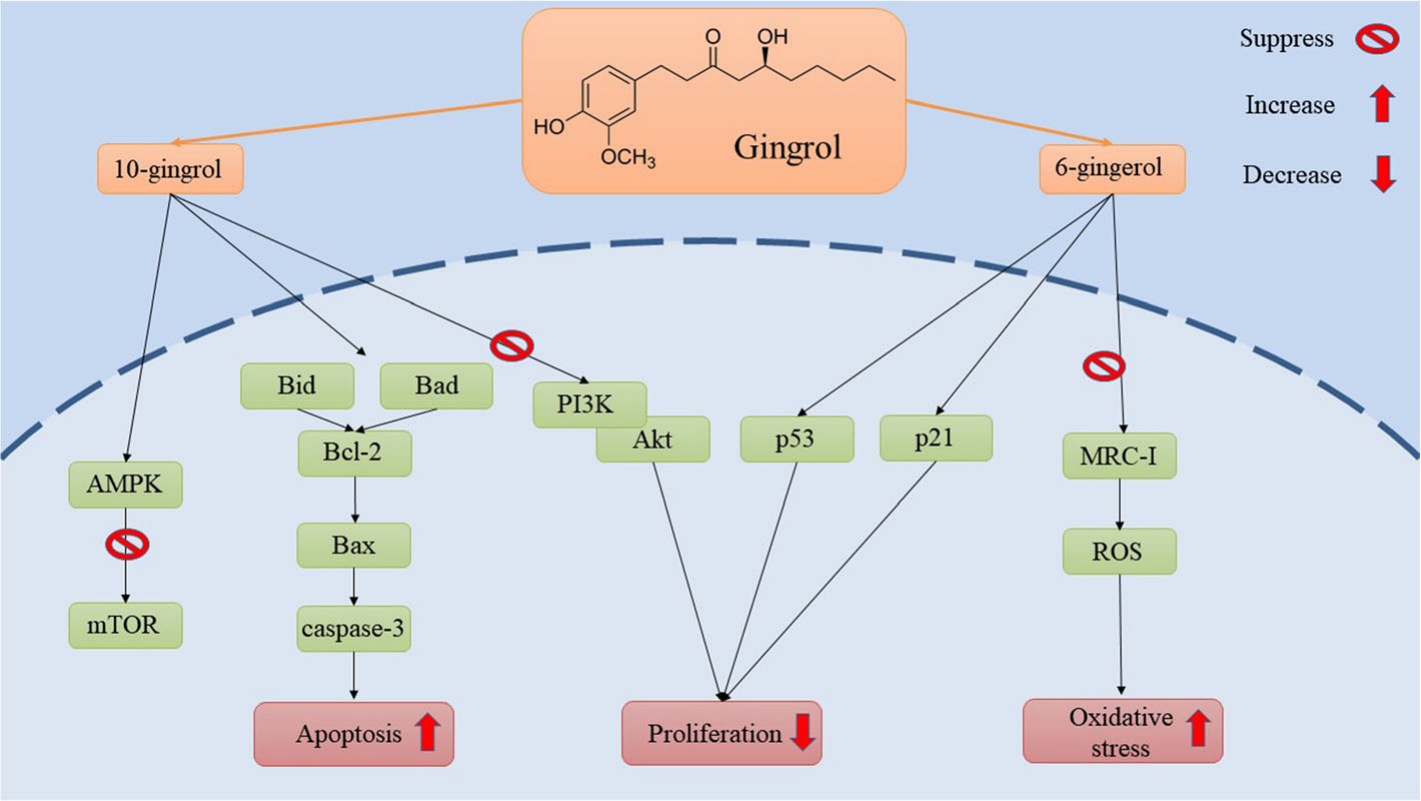
Schematic representation of gingerol applications on signaling pathway in cervical cancer

Cervical cancer is one of the most common and important gynecological cancers, which has become a global concern with an increasing number of patients and mortality rates. Although surgery, chemotherapy or radical hysterectomy, and radiotherapy are effective treatments for this disease, the side effects of these methods endanger a person’s quality of life and cause other problems in other parts of the body. In this regard, the establishment of new methods with fewer side effects seems to be necessary. Ginger is one of the plants with valuable compounds such as gingerols, paradols and shogoals, which is a rich source of antioxidants, anti-cancer and anti-inflammatory agents. Studies show that ginger by participating in various signaling pathways, can play a significant role in the prevention and treatment of cervical cancer (Fig. 1). Apoptosis, cell proliferation, altered cell morphology, suppressing proteasome and reactivating p53, DNA damage, redox potential regulation, and the production of free radicals are examples of pathways that ginger uses to treat cervical cancer.

Induction of apoptosis and inhibition of cell proliferation are the main routes through which ginger restricts the progression of cervical cancer and can cure it. Also, inhibiting miR-629 expression and inhibiting the production of prostaglandins and inhibiting the PI3K/Akt pathway are other ways in which ginger extract improves cervical cancer. After all, it seems that ginger can be a safe natural treatment for cervical cancer. On the other hand, by combining ginger with other treatments such as the use of chemotherapy drugs, more effective treatment with fewer side effects can be achieved. Since there are not enough studies to determine the appropriate and effective dose of this plant, more clinical studies inside the body and in the laboratory, it is necessary for the therapeutic purposes of this plant. For instance, the effect of gingerol on cancer stem cells which are responsible for many tumor features such as drug resistance is not investigated.

We suggest that also using nanotechnology for enhancing the effects of gingerol, improving bio viability, monitoring its release, lowering its side effects, and finally providing a more personalized treatment should be considered by future researches. Additionally, despite the advantages of herbal medicine there are some limitations for using these products against cancer. For instance, all of the data on the efficacy of herbal compounds might not be true due to some impurities which have their own biological activity. This means that still more precise investigations are required for approving the effects of these compounds and their safeness. Moreover, in spite of the general belief, herbal medication is not completely safe and has also some side effects including nephrotoxicity [157]. In this regard, the following measurements are needed before using herbal medication in clinics: detection and characterization of every bioactive ingredient of the intended herbal compound and the standardization of these extracts.

## Data Availability

Not applicable.

## Abbreviations

HPV: human papillomavirus
iNOS: Inducible nitric oxide synthase
Apaf-1: Apoptotic protease activating factor 1
MRC I: Mitochondrial respiratory complex I
H2AX: H2A histone family member X.
ER: Endoplasmic reticulum
PARP: Poly ADP ribose polymerase
PERK: Protein kinase RNA-like endoplasmic reticulum kinase
RSU-1: Ras suppressor-1
PGD2: Prostaglandin D2
PGE2: Prostaglandin E2
PGF2: Prostaglandin F2
PGI2: Prostaglandin I2
COX: Cyclooxygenase
ROS: Reactive oxygen species
DR3: Death receptors 3
DR5: Death receptors 5
PKCε: Protein kinase Cε
NF-kB: Nuclear factor-Kappa B
